# Assessing the feasibility of single target radiosurgery quality assurance with portal dosimetry

**DOI:** 10.1002/acm2.12578

**Published:** 2019-04-01

**Authors:** Elizabeth L. Covington, Jesse D. Snyder, Xingen Wu, Rex A. Cardan, Richard A. Popple

**Affiliations:** ^1^ Department of Radiation Oncology University of Alabama – Birmingham South Birmingham AL USA

**Keywords:** portal dosimetry, quality assurance, stereotactic radiosurgery

## Abstract

**Purpose:**

To assess the feasibility of using portal dosimetry (PD) for pre‐treatment quality assurance of single target, flattening filter free (FFF), volumetric arc therapy intracranial radiosurgery plans.

**Methods:**

A PD algorithm was created for a 10X FFF beam on a Varian Edge linear accelerator (Varian Inc, Palo Alto, CA, USA). Treatment plans that were previously evaluated with Gafchromic EBT‐XD (Ashland, Bridgewater, NJ, USA) film were measured via PD and analyzed with the ARIA Portal Dosimetry workspace. Absolute dose evaluation for film and PD was done by computing the mean dose in the region receiving greater than or equal to 90% of the max dose and comparing to the mean dose in the same region calculated by the treatment planning system (TPS). Gamma analysis with 10% threshold and 3%/2 mm passing criteria was performed on film and portal images.

**Results:**

Thirty‐six PD verification plans were delivered and analyzed. The average PD to TPS dose was 0.989 ± 0.01 while film to TPS dose was 1.026 ± 0.01. All PD plans passed the gamma analysis with 100% of points having gamma <1. Overall, PD to TPS dose agreement was found to be target size dependent. As target size decreases, PD to TPS dose ratio decreased from 1.004 for targets with diameters between 15–31 mm and 0.978 for targets with diameters less than 15 mm.

**Conclusion:**

The agreement of PD to TPS mean dose in the high dose region was found to be dependent on target size. Film measurements did not exhibit size dependence. All PD plans passed the 3%/2 mm gamma analysis, but caution should be used when using PD to assess overall dosimetric accuracy of the treatment plan for small targets.

## 1 INTRODUCTION

Intracranial stereotactic radiosurgery (SRS) is used to precisely deliver a high dose of radiation to targets within the brain with a single treatment.^1^, ^2^, ^3^ Due to high accuracy positional and dosimetric accuracy needed for a successful treatment, pre‐treatment quality assurance (QA) is vital step in ensuring safe delivery.^4^ Selection of an appropriate QA device is complicated by the detector size vs field size and loss of lateral charged particle equilibrium.^3^ Recommendation are to use an appropriate dosimeter with a resolution of approximately 1 mm of better.^5^

Portal dosimetry is commonly used in the pre‐treatment QA for intensity‐modulated plans^6^, ^7^, ^8^, ^9^, ^10^, ^11^ by comparing the delivered fluence on the portal imager to that predicted by the treatment planning system (TPS). This is done on the Varian (Varian Medical Systems, Palo Alto, CA, USA) platform through the creation of a Portal Dosimetry Image Prediction (PDIP) algorithm that enables users to create a predicted portal dose image for fields with dynamic multileaf collimators (MLCs) and compare the acquired image in the Portal Dosimetry workspace. While the technique has been used often for traditional treatments, its use in small field SRS treatments has been less explored in the literature.^12^ Because of the greater potential harm with these treatments, care should be taken to validate the dosimetric performance of the device for appropriate field shapes, sizes, total doses, and dose rates.

This study focuses on assessing the feasibility of using portal dosimetry for pre‐treatment QA of 10 MV flattening filter free (10X FFF), volumetric arc therapy single target SRS treatment plans on an Edge linear accelerator (Varian, Palo Alto, CA, USA) with a high definition MLC (HDMLC). The Edge linear accelerator employs a 43 × 43 cm^2^ Digital Megavolt Imager (DMI) with an image size of 1280 × 1280 pixels which results in submillimeter resolution of images. A PDIP algorithm was created and used to compare the results of portal dosimetry pre‐treatment QA to our departmental standard of film.

## 2 METHODS

Clinical treatment plans were chosen that had been previously evaluated with Gafchromic EBT‐XD (Ashland, Bridgewater, NJ, USA) film. The dose distribution was measured in the coronal plane using radiochromic film (EBT‐XD, Ashland Chemical, Covington, KY, USA). A calibration curve was obtained at each measurement session. The film was scanned using an Epson model V700 PhotoPerfection document scanner (Epson America, Long Beach, CA, USA) and converted to dose using a three‐channel technique.^13^ Film analysis was done using in house software developed in MATLAB (MathWorks, Natick, MA, USA). Gamma analysis was done with a 10% threshold and 3%/2 mm criteria.^14^ Absolute dose evaluation was done by computing the mean dose in the region receiving greater than or equal to 90% of the max dose and comparing to the mean dose in the same region calculated by the TPS. The mean dose in this region‐of‐interest was used rather than a point dose to reduce the effects of pixel‐to‐pixel noise in the film.^15^ Plans were chosen to cover a range of target sizes from 3 to 31 mm equivalent diameter, which is the diameter of a sphere with the same volume as the target. All analysis was performed on the composite dose of the two arcs. The median prescription dose per fraction was 15 Gy, with a range of 5–20 Gy.

Portal dosimetry was commissioned on an Edge linear accelerator 10X FFF beam with HDMLCs. Varian does not offer a preconfigured package for machines with HDMLCs and/or FFF beams. In order to create a predicted portal image, a PDIP algorithm must be created by the user. This was done by following the instructions provided by Varian in the *Eclipse Photon and Electron Algorithms Reference Guide*. Once the algorithm was created, it was verified using the verification plans provided in the preconfigured PDIP package for flattened beams with Millennium MLCs. These plans were imported, the MLC files were updated for HDMLCs, the energy was changed to 10X FFF, and the plans were recalculated.

Plans were delivered on an Edge linear accelerator with a DMI portal imager at 100 SDD and evaluated in the Portal Dosimetry workspace. All plans were delivered with the planned dose rate of 2400 MU/min. Since the portal imager has a fixed geometry with the gantry, couch rotations are not included in the portal dosimetry delivery, hence the delivery is perpendicular composite delivery rather than a true composite delivery like film.^14^ Like the film measurements, the analysis was performed on the composite dose of the two arcs. Gamma analysis was done in absolute mode with 10% threshold and 3%/ 2 mm passing criteria. The Portal Dosimetry workspace tools do not allow dose thresholding above 80% or dose comparison of a specified region, so absolute dose analysis was done through the Portal Dosimetry scripting tool. A script was developed to recreate the process used for film analysis. Absolute dose evaluation was done by computing the mean dose in the region receiving greater than or equal to 90% of the max dose in the portal image and comparing to the mean dose in the same region in the predicted portal image.

**Figure 1:**
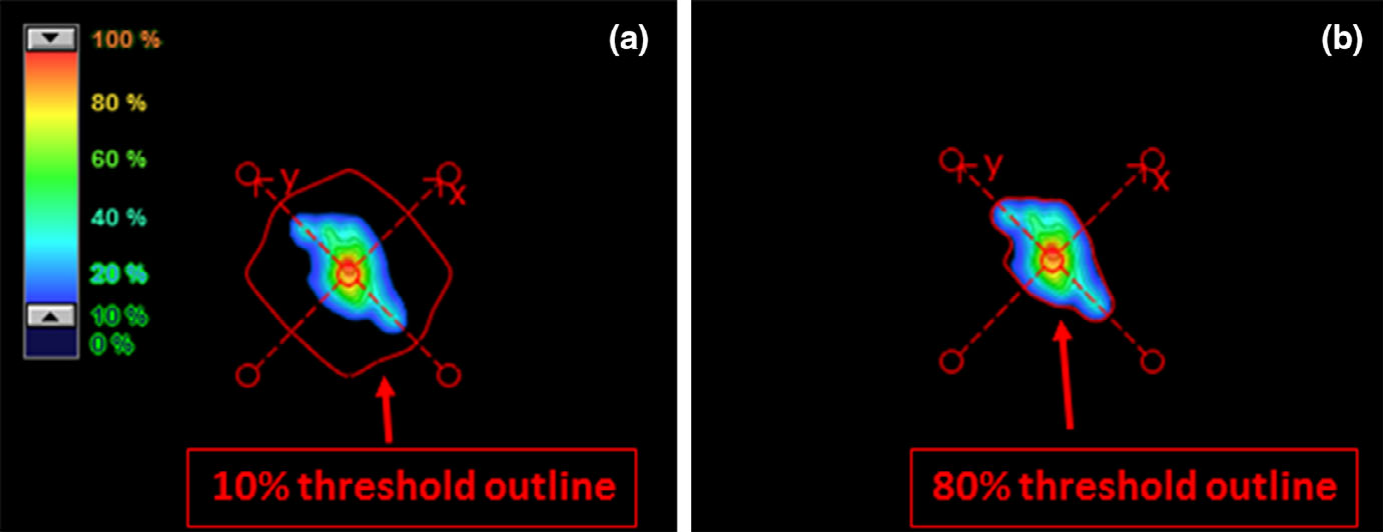
(a) Color wash shows 10% dose and greater while red outline shows the 10% dose threshold set by portal dosimetry workspace which clearly does not match due to the exclusion of high signal pixels. (b) Threshold was manually adjusted to approximately 80% to match the 10% isodose line to desired analyzed region.

The gamma analysis of portal images was initially done with a 10% dose threshold until an issue was observed where the region of interest outline did not match the 10% dose color wash, as shown in Fig. 1(a). The red outline in Fig. 1(a) shows the 10% threshold on a 3 mm target that is significantly larger than the 10% dose color wash. Once the analysis threshold was set to 80% in Fig. 1(b), the outlines match. The Portal Dosimetry workspace thresholds are limited by a hard coded gamma histogram cut‐off that excludes high signal pixels in order to prevent them from being used as the global gamma reference value. In order to perform the gamma analysis with a true 10% threshold, the 10% isodose line was displayed on the composite dose. The threshold was manually adjusted until the dose distribution matched the desired isodose line. After setting the desired threshold, a 3%/2 mm gamma analysis was performed. The results of the two gamma analyses were compared.

The effects of the high dose rate on the agreement between the predicted and delivered portal images was also investigated by assessing the agreement of various open fields at dose rates of 400, 1200, and 2400 MU/min. Portal dosimetry requires dynamic MLCs for creating predicted images; therefore, deliverable open fields were created by setting MLCs completely open and having the field shaped only by jaws. One MLC leaf was moved approximately 1 mm behind the jaws to make the plan dynamic and enable the creation of a predicted portal image.

## 3 RESULTS

Commissioning of portal dosimetry showed no field size dependence, as shown in Table 1. Two dynamic plans, Aria and DynChair, that were included in the preconfigured Truebeam package were also measured, and had gamma passing rates of 100% and 99.7% with a 10% dose threshold and 3%/2 mm criteria.

**Table 1:** Percent difference of portal dose image prediction (PDIP) vs measured calibration units (CU) for various field sizes in portal dosimetry

Field Size (cm^2^)	Measured (CU)	PDIP (CU)	% Difference
2 × 2	44.6	45.3	−1.6
3 × 3	92.9	94.4	−1.6
5 × 5	96.0	97.6	−1.7
10 × 10	99.1	100.5	−1.4
15 × 15	100.4	101.8	−1.4
20 × 20	101.3	102.7	−1.4
30 × 20	101.3	103.0	−1.6

**Figure 2:**
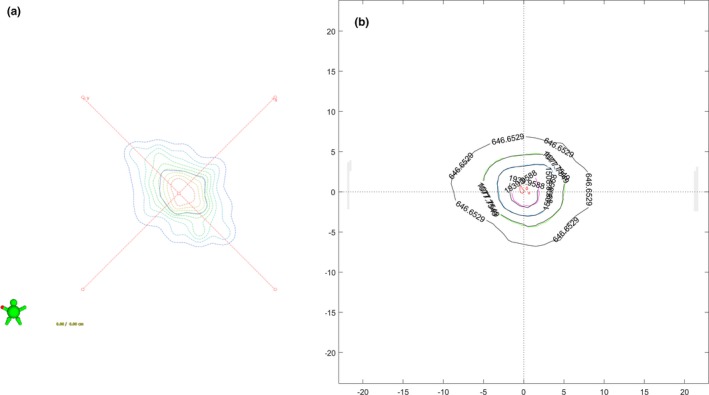
(a) Perpendicular composite dose of a two arc plan in portal dosimetry. (b) True composite dose measured with film and analyzed in MATLAB. Note that the shape difference is due to the fixed geometry of the portal imager and its inability to deliver non‐coplanar beams.

Thirty‐six clinical plans were analyzed with portal dosimetry. Target sizes ranged from 3 to 31 mm equivalent diameter with an average of 19 mm. Thirty‐five plans contained two noncoplanar arcs with one arc at table angle 0° (IEC coordinate system). The table angle of the other arc was 70° (15 plans), 290° (7 plans), 80° (5 plans), 300° (4 plans), 50° (1 plan), 60° (2 plans), or 280° (1 plan). One plan contained two coplanar arcs at table angle zero. Figure 2 shows an example of composite dose from a two arc treatment of a 5.4 mm diameter target in portal dosimetry and film. The difference in the dose distribution is due to delivering the film with all planned couch rotations (i.e., true composite) while the portal imager is only able to deliver coplanar beams with respect to the imager (i.e., perpendicular composite).^14^

All portal dosimetry plans were analyzed in absolute dose with gamma analysis criteria of 3%/2 mm and 10% dose threshold per recommendations of AAPM Task Group No. 218.^14^ All portal dosimetry plans passed with 100% of the points passing the gamma criteria. Plans were also analyzed with our department criteria of 3%/1 mm, since a 2 mm offset was deemed clinically unacceptable due to our SRS plans have a zero margin PTV (i.e., GTV = PTV).^16^, ^17^ For 10% threshold and 3%/1 mm, the average percentage of points passing was 99.95%. The gamma analysis on the portal images was performed again after manually adjusting the threshold until region of interest matched the 10% color wash and the average gamma passing rate was 99.99% for 3%/2 mm passing criteria. For film, the average percentage of points passing was 98.89% for 10% dose threshold and 3%/2 mm criteria and 97.14% for 3%/1 mm.

**Figure 3:**
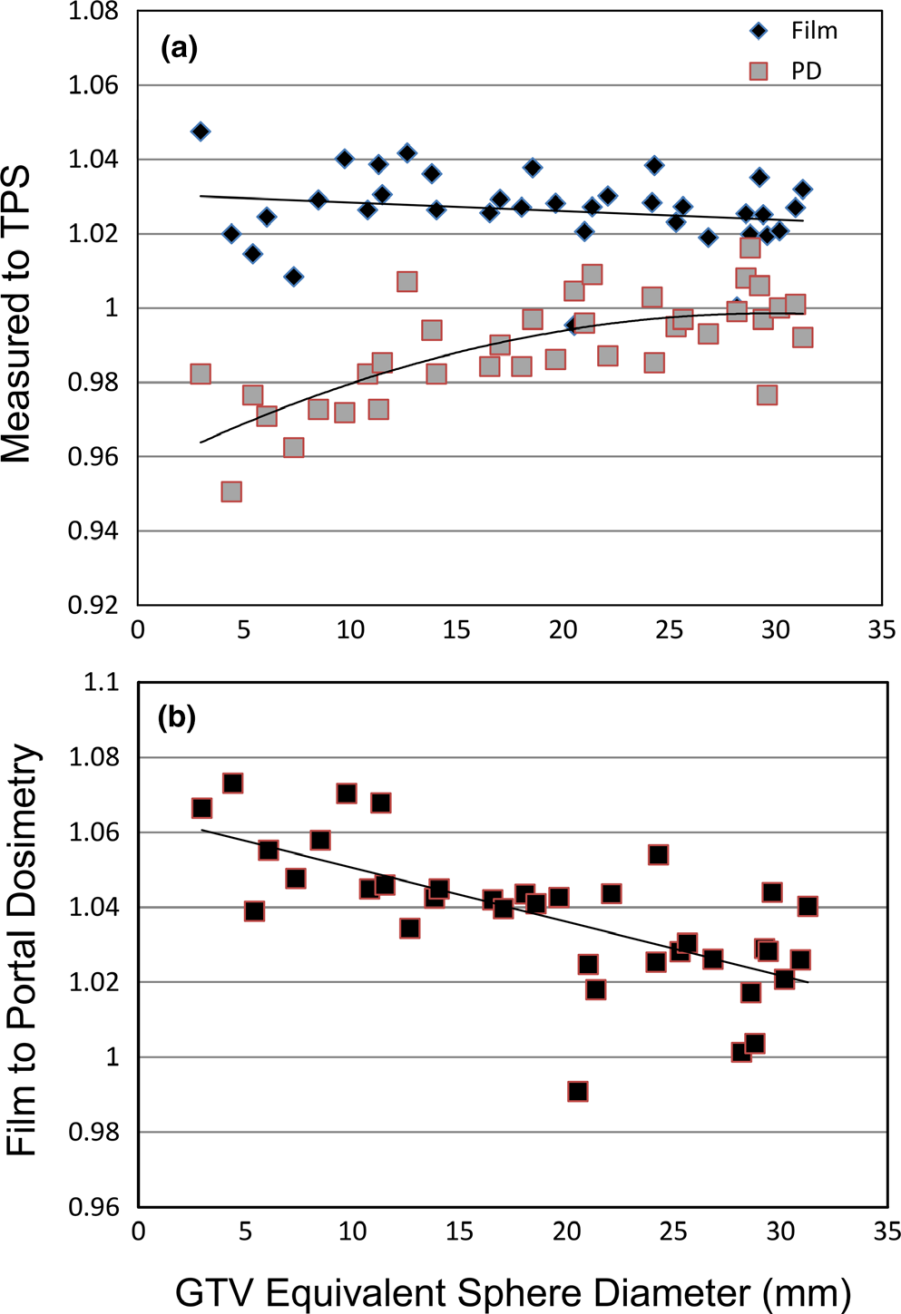
(a) The measured to treatment planning system (TPS) dose in the >90% maximum dose region as a function of target size. While the film remains relatively flat across all target size, the portal dosimetry results are target size dependent. (b) The ratio of film to portal dosimetry measurements as a function of target size.

The average film to TPS dose in the high dose region was 1.026 ± 0.01 (range 0.995–1.048) while the measured portal image to PDIP was 0.992 ± 0.02 (range 0.951–1.016). Figure 3(a) shows the measured to TPS or PDIP dose as a function of target size. Figure 3(b) shows the film/TPS to PD/PDIP ratio as a function of target size.

**Figure 4:**
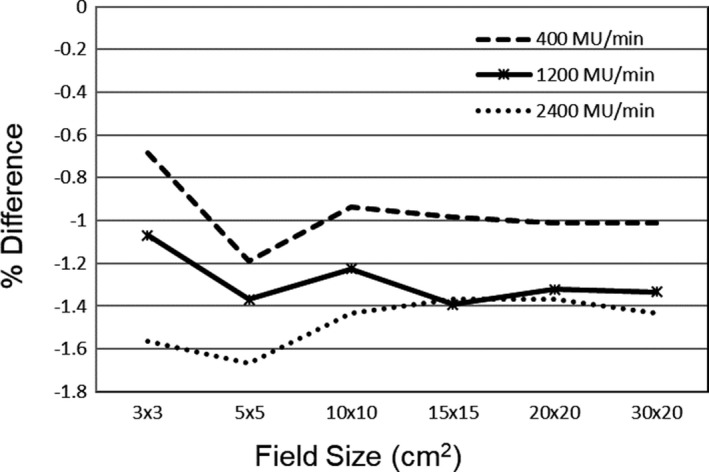
The percent difference between the delivered and predicted portal image output at central axis for open fields at dose rates of 400, 1200, and 2400 MU/min.

Open fields of size 3 × 3–30 × 20 cm^2^ were delivered at 400, 1200, and 2400 MU/min to assess any dose rate dependence of the DMI imager. As shown in Fig. 4, the lowest dose rate showed the greatest agreement with the predicted image. The clinically used dose rate showed an increased difference at smaller field sizes.

## 4 DISCUSSION

While all plans evaluated with portal dosimetry passed our department criteria of 5% absolute dose difference, there was an offset between the normalization factors measured via film, as shown in Fig. 3. For targets with an equivalent target diameter greater than 15 mm, the average ratio of film to portal dosimetry was 1.03. For targets with an equivalent diameter less than 15 mm, this ratio increased to over 1.05 and approached 1.08 for the smallest targets measured. PD commissioning results did not show a field size dependence, and the dose rate effects for open beams showed a decrease in the measured plan at the highest dose rate. This dose rate effect could be part of the overall offset observed between the portal and film, since Gafchromic film has been shown to have a minimal dose rate response.^18^ Xu et al. found that the gamma passing rate decreased from 99.9% to 91.5% when the dose rate was increased from 400 to 1200 MU/min for 10X FFF beams on a DMI imager.^19^ While decreasing the dose rate may improve the PD analysis, it is not recommended since ASTRO guidelines^20^ state that “same files to be used for the patient delivery should be used for the QA measurements.”

Ballangrud^12^ et al. reported on using portal dosimetry to validate multi‐lesion (range 2–8) SRS plans. Their SRS beam model was created and validated using five preclinical plans and adjusting the beam model until film and portal dosimetry measurements agreed within 3% using gamma pass criteria of 95% for 3%/2 mm and a 10% threshold. They noted that some clinical plans required a dose threshold of 25% to meet their gamma pass criteria. Rather than adjust our beam model, we used our film measurements as a benchmark to evaluate portal dosimetry. Note that that all of our PD plans passed using the gamma criteria. Only the comparison of dose (or CU) in the high dose region revealed shortcomings in the portal dosimetry workflow. We also chose to evaluate portal dosimetry using single target plans to avoid convolving issues due to multiple targets and blurring of the dose distribution due to the fixed geometry of the gantry with respect to the imager.

All departmental clinical decision on the acceptability of SRS treatment plans are based on the absolute dose difference between the calculated and delivered plan rather than the results of gamma analysis. While gamma analysis provides information about overall dosimetric and positional accuracy, this information cannot be separated to provide the physicians with an overall description of how “hot” or “cold” the plan delivered, which is the leading factor for re‐planning. When relying on gamma analysis for plan evaluation, picking the appropriate gamma criteria is an ongoing challenge in our field and shortcomings of its use has been previously published.^21^, ^22^ While scripting allowed us to overcome the shortcomings of the Portal Dosimetry analysis tools, the overall offset between the film and portal dosimetry analysis, especially with respect to small target sizes, provides challenges for using Portal Dosimetry as the sole tool for the analysis of stereotactic treatment plans.

While portal dosimetry provides a quick method for checking the overall integrity of MLC motions and gross error, it is not capable of providing an absolute dose comparison. The plans are presented in Calibration Units (CU), which are determined by a calibration procedure of exposing the imager to a 10 × 10 cm^2^ and entering the delivered dose. This is used to do an “absolute dose” comparison within the portal dosimetry workspace which is used within the gamma analysis. The current version of the software is unable to isolate the dose comparison from the gamma analysis. The hard coded histogram cutoff also provides challenges for analysis. Currently, the histogram cutoff can be set up to 5% to remove the highest signal peaks to prevent the inclusion of defective pixels. This setting is applied to the entire 1280 × 1280 image and not within the pixels where the dose is contained. This affects the thresholding of the image for the gamma analysis and leads to the inconsistencies seen in Fig. 1 which is more pronounced for smaller targets. We caution users to test the accuracy of the set thresholds in portal dosimetry before using the analysis to make clinical decisions.

Portal imagers can also undergo radiation damage that affects the accuracy of measurements. Ritter et al.^23^ reported a technique to measure a dosimetric leaf gap surrogate, called the leaf offset constancy (LOC) using an EPID. In this study, irregularities were seen in LOC measurements that were attributed to radiation damage after repeated exposure to an FFF beam. The damage was not mitigated by dark and flood field calibrations. Due to this, our observed dose rate dependencies, and inconsistencies between our film and EPID measurements, caution should be used when using portal images to evaluating treatment plans with small targets. Furthermore, portal dosimetry should not be used for the commissioning and validating of stereotactic beam models.

## 5 CONCLUSION

Portal dosimetry measurements were found to be target size dependent and could deviate up to 8% from film measurements for the smallest targets evaluated. While portal dosimetry provides a quick method to evaluate SRS plans for gross error without the use of a specialized phantom, it does not provide an accurate method for determining the dosimetric accuracy of the plan when compared to film. Due to the fixed geometry of the portal imager with the gantry, it is also unable to evaluate the accuracy of the true dose distribution due to the inability to deliver couch rotations. Overall, caution should be used when using PD to assess overall dosimetric accuracy of stereotactic treatment plans and commissioning stereotactic beam models.

## CONFLICT OF INTEREST

The authors have no conflict to disclose.
